# Impact of Acute Insulin Resistance on Myocardial Blush in Non-Diabetic Patients Undergoing Primary Percutaneous Coronary Intervention

**DOI:** 10.3389/fcvm.2021.647366

**Published:** 2021-05-10

**Authors:** Soheir M. Kasem, Ghada Mohamed Saied, Abdel Nasser MA Hegazy, Mahmoud Abdelsabour

**Affiliations:** ^1^Department of Internal Medicine, Faculty of Medicine, Assiut University, Assiut, Egypt; ^2^Department of Clinical Pathology, Faculty of Medicine, Assiut University, Assiut, Egypt; ^3^Department of Cardiology, Assiut University, Assiut, Egypt

**Keywords:** acute insulin resistance, myocardial blush, non–diabetics, Primary PCI, Acute coronary syndromes

## Abstract

**Background:** Myocardial blush grading is considered to be a novel tool for assessment of coronary microvasculature and myocardial perfusion in patients undergoing coronary angiography and angioplasty, and its reduction identifies patients at high risk. Our study aimed to evaluate the association between acute insulin resistance and myocardial blush in non-diabetic patients with ST-segment elevation myocardial infarction (STEMI).

**Methods:** Two hundred forty non-diabetic patients with STEMI who underwent primary percutaneous coronary intervention were consecutively recruited. The relationship of homeostasis model assessment—estimated insulin resistance (HOMA-IR) to myocardial blush and in-hospital outcome was investigated.

**Results:** Higher HOMA-IR tertile was observed in obese patients, with hyperinsulinemia, had Killip class >1, with higher CPK-MB level and was correlated to impaired myocardial blush after adjusting for the other confounding risk factors. It was also concluded that higher HOMA-IR was independently associated with no/minimal myocardial blush after STEMI. Moreover, it was founded to be an independent predictor of pulmonary edema and impaired left ventricular systolic function.

**Conclusion:** This study revealed that acute insulin resistance was prevalent in non-diabetic patients with STEMI and was an independent predictor for post-infarction myocardial and microvascular injury and poor in-hospital outcome.

**Trial Registration:** The trial was registered at the registry of Clinicaltrials.gov, ClinicalTrials.gov Identifier: NCT04651842, Date of registration: 2^nd^ December 2020 Registry URL, https://clinicaltrials.gov/ct2/show/NCT04385589?cond=Dapagliflozin+in+diabetic+patients&cntry=EG&draw=2&rank=1.

## Background

Complete myocardial reperfusion and restoration of coronary microcirculatory function (CMF) is a therapeutic goal in ST-segment elevation myocardial infarction (STEMI) (1). However, the success of primary percutaneous coronary intervention (pPCI) is not achieved in 30% to 50% of patients (2, 3).

It was found that insulin resistance (IR) plays substantial role in the development of cardiac-vascular infirmities and carries bad prognostic outcome for acute myocardial infarction (AMI) (4).

IR was found to be related to cardiac injuries on both muscular and microvascular levels after STEMI. Likewise, after adjusting for other integrals of metabolic syndrome, IR was associated with myocardial injury after elective PCI (5).

Now, IR was assessed by the homeostatic model assessment (HOMA) index in the early phase of acute coronary syndrome in non-diabetic patients. This “acute IR,” considered a part of the acute glycol-metabolic response to stress, may be transient and also can occur even in patients without chronic glycol-metabolic derangements (6).

Acute IR includes acute hyperglycemia and/or acute hyperinsulinemia. Hyperglycemia has the importance of prognostic relevance of hyperinsulinemia in STEMI patients, but its relationship with coronary flow is still unclear (7, 8). However, the direct acute negative cardiovascular effects of hyperinsulinemia is acknowledged as it is contributing to incomplete myocardial reperfusion and CMF impairment (8).

Myocardial blush is a qualitative visual assessment of the amount of contrast medium filling a territory supplied by a pericardial coronary artery (9) (was first defined by Arnoud van't Hof et al.). Myocardial blush grade (MBG) is considered a reliable and valid tool for assessing coronary microvasculature and myocardial perfusion in patients undergoing coronary angiography and angioplasty (10). Decreased blush grade was used to identify those at increased risk who require intensive management both during the procedure to improve myocardial perfusion and later for secondary prevention (10).

The current study hypothesis was that acute IR can occur in the early post pPCI period even in non-diabetic patients as a dynamic phenomenon, and it could be related to the development of microvascular injury. Myocardial blush is defined as a marker of coronary microvascular function; accordingly, IR was evaluated in relation to myocardial blush in nondiabetic STEMI patients treated by pPCI as a primary endpoint. The residual ST-segment elevation, post-TFC% and major adverse cardiovascular events (MACE) were secondary endpoints. The HOMA index is a simple and inexpensive marker of IR, primary used in chronic states. It was recently validated in STEMI patients as feasible for assessing IR during myocardial infarction and therefore used in the current study (11).

## Methods

### Study Participants

This cross-sectional comparative study included 240 non-diabetic patients with acute STEMI selected from the Cardiology Department, Assiut University Hospital, who underwent primary percutaneous coronary intervention (PCI) between May 1, 2018 and May 1, 2019. Sample size was calculated using the G^*^Power 3 software. With a power of 95% and type I error of 5% (α = 0.05 and β = 95%), the minimum required sample was 210 patients (further divided into three equal groups according to HOMA-IR tertiles) for an effect size of 10% in rate of myocardial blush grade.

Exclusion criteria were diabetic patients, renal insufficiency, advanced hepatic dysfunction, had malignancy, chronic heart failure/cardiomyopathy, prior myocardial infarction, and diseases requiring steroid therapy; patients on antioxidant supplement/therapy within 4 weeks before enrolment in the study, pregnant females, alcoholic, patients allergic to radiographic contrast, and uncooperative/refusal.

Ethical approval was obtained from the Medical Faculty, Assiut University (IRB No. 17300510) and was adherent to the guidelines of the declaration of Helsinki.

### Clinical and Laboratory Assessment

After obtaining informed consent, the included patients were subjected to:

1. Proper history taking including history for the presence of cardiovascular risk factors: hypertension, diabetes mellitus, dyslipidemia; smoking and family history of premature coronary artery disease, history of ischemia, previous CCU admission, and previous PCI.
2. Complete physical examination including body mass index (BMI) was calculated using the formula of weight/height^2^ (kilograms per square meter).
3. Blood pressure was measured in a seated position after a 10-min rest, using an electronic blood pressure monitor (Microlife AG, 9443 Widnau, Switzerland).
4. Diagnosis of diabetes was made according to the criteria of the American Diabetes Association, and prediabetes was defined by fasting blood glucose of 100 mg to <126 mg/dl, 2-h plasma glucose of 140 to <200 mg/dl, or HbA1C of 5.7 to <6.5% (12).
5. Hypertension was diagnosed according to the 8th Report of the Joint National Committee on Prevention, Detection, Evaluation, and Treatment of High Blood Pressure (JNC_8), and hyperlipidemia was diagnosed according to the guidelines of the National Cholesterol Education Program (ATP III) (13, 14).
6. Blood samples were collected in an air-conditioned and quiet room. Serum glucose, blood urea, creatinine, total cholesterol, low-density lipoprotein—cholesterol (LDL-C), high-density lipoprotein—cholesterol (HDLC), and triglycerides were assessed using a HITACHI 912 Analyzer (Roche Diagnostics, Germany). Insulin and creatine kinase MB isoform level were assessed by AIA 900, fluorescence enzyme immunoassay (FEIA) method (Japanese).

HOMA-IR was calculated according to the formula (fasting insulin in mIU/L × fasting glucose in mg/dl)/405^15^. Normal reference levels for HOMA-IR range between 0.7 and 2.0. HOMA-IR is a preferred estimate for insulin resistance as glucose clamp methods, the current gold standard, are resource intensive and time consuming. Several studies use 2.0 as cutoff for increased insulin resistance (15).

### Angiographic Assessment

After the angioplasty procedure immediately, epicardial and myocardial reperfusion was assessed and graded on the angiograms. In each patient, the best projection was chosen to assess the myocardial region of the infarct-related coronary artery, preferably without super positioning of non-infarcted myocardium. Angiographic runs must be long enough to allow some filling of the venous coronary system, and backflow of the contrast agent into the aorta have to be present to be sure of adequate contrast filling of the epicardial coronary artery.

All angiograms will be made after 400 μg of nitroglycerin intracoronary has been given immediately after the primary angioplasty procedures, and this procedure allows quantitative coronary artery analysis (16): First, epicardial reperfusion is assessed by thrombolysis in myocardial infarction (TIMI) flow grades (TFGs) as the following: Grade 0 means no perfusion, Grade 1 means penetration without perfusion, Grade 2 means partial perfusion, Grade 3 means complete perfusion (17). Epicardial reperfusion was also assessed by thrombolysis in myocardial infarction (TIMI) frame count (TFC), which is defined as the number of frames required for dye to first opacify a standard distal landmark (18). Second, myocardial reperfusion is assessed by TIMI myocardial perfusion grade (TMP) as the following: TMP Grade 0 denotes failure of dye to enter the microvasculature, TMP Grade 1 denotes dye slowly enters but fails to exit the microvasculature, TMP Grade 2 denotes delayed entry and exit of dye from the microvasculature, TMP Grade 3 denotes normal entry and exit of dye from the microvasculature (19).

Myocardial reperfusion had been also determined by measuring ST-segment resolution (STR) analysis so ECGs had been done on admission (first ECG) and 90 min after the primary PCI in the coronary care unit (second ECG). The second ECGs had been classified with regard to the ST segment resolution into the following grades: Normalized, defined as no residual ST segment elevation; Improved, defined as a residual ST segment elevation <70% of that on the first ECG; Unchanged, defined as a residual ST segment elevation > 70% of that on the first ECG (20).

For all patients, postprocedural transthoracic echocardiography left ventricular ejection fraction was measured by biplane Simpson method using the end diastolic and end systolic apical four- and two-chamber views for estimation of left ventricular volume and calculation of ejection fraction. In hospital primary angioplasty procedures, major adverse cardiovascular events (MACEs) were determined and defined as the composite of death, stent thrombosis, re-infarction, cardiogenic shock, and stroke (20).

Data entry, verification, and validation were carried out by the researcher, and analyses were performed via IBM-SPSS software (Statistical Package for the Social Sciences, version 24, SSPS Inc, Chicago, IL, USA). Numerical data were expressed as mean ± SD, and frequency tables with percentages were used for categorical variables. χ^2^ and Monte Carlo exact test was used to compare the difference in the distribution of frequencies among different groups. One-way ANOVA was used to determine the mean difference between groups. Binary logistic forward regression analysis was used to define the independent predictors of MBG variables that showed significant differences among the studied groups. A value of *p* < 0.05 was considered statistically significant.

## Results

**Figure 1:**
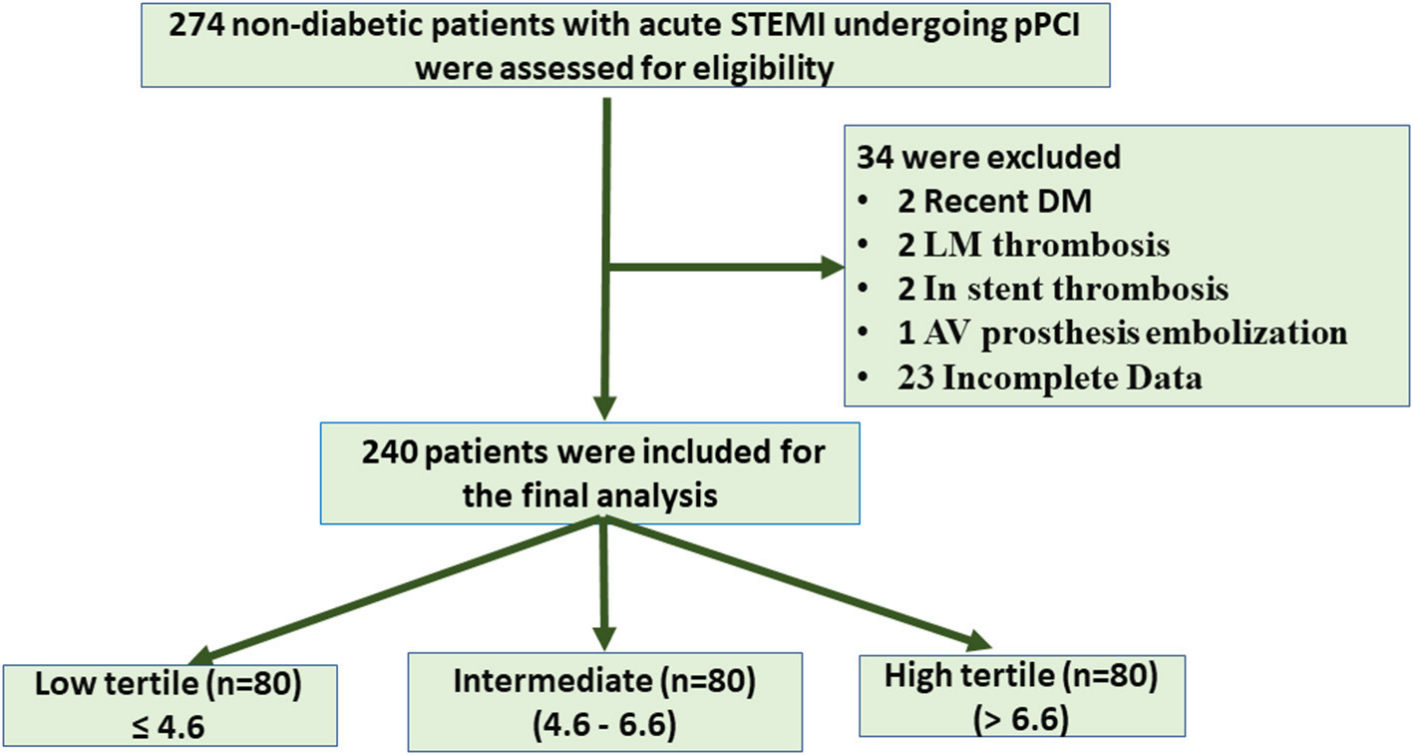
Flow chart of patient enrollment. STEMI, ST segment elevation myocardial infarction; PCI, primary coronary intervention; LM, left main; AV, aortic valve.

This study aimed to evaluate the impact of insulin resistance in non-diabetic (IR) on myocardial blush and in hospital MACE in the setting of STEMI patients treated with PCI. Non-diabetic patients (274), with anterior and non-anterior STEMI, who were admitted to the ICU during the study period, were recruited. Thirty-four patients were excluded for recently discovered diabetes mellitus, left main thrombosis, aortic valve prostheses with embolization, stent thrombosis, and defect of data counting. Hence, 240 patients (classified based on equal tertile of HOMA IR level into three equal groups (low ≤ 4.6, intermediate 4.6–6.6, and high > 6.6) were available for analysis (Figure 1).

The majority of the studied patients were males aged >50 years with insignificant difference between groups in relation to HOMA-IR. Moreover, acute insulin resistance as estimated by HOMA-IR tertile was significantly higher in obese, those with heart failure (Killip class >1), and higher plasma insulin level, CPK-MB, and fasting blood glucose level (Table 1).

**Table 1:** Baseline demographic and clinical correlates of HOMA_IR.

**Parameter**	**Low** ** (*n* = 80)**	**Intermediate** ** (*n* = 80)**	**High** ** (*n* = 80)**	***P*-value**
Age/years	51.98 ± 10.2	52.95 ± 12.5	52.76 ± 12.3	= 0.855^*^
Sex (Male)	70 (87.5%)	63 (78.8%)	63 (78.8%)	= 0.256^**^
Smoker	51 (63.8%)	48 (60%)	52 (65%)	= 0.793^**^
Obesity	36 (45%)	40 (50%)	45 (56.3%)	**=** 0.039^**^
Hypertensive	12 (15%)	23 (28.8%)	20 (25%)	= 0.093^**^
Killip class > 1	9 (11.3%)	7 (8.8%)	14 (17.5%)	**=** 0.044^**^
GP IIb/IIIa Inhibitor	29 (36.3%)	32 (40%)	33 (41.3%)	= 0.518^**^
Pain-Balloon Time	4.24 ± 0.3	5.79 ± 0.6	4.19 ± 0.4	**=** 0.035^*^
FBG	98.51 ± 6.6	99.33 ± 8.5	101.19 ± 11.3	**=** 0.028^*^
Insulin Level	12.38 ± 0.5	23.03 ± 3.1	34.47 ± 7.5	**<** 0.001^*^
LDL	105.14 ± 23.2	108.11 ± 22.1	104.47 ± 18.9	= 0.642^*^
HDL	46.85 ± 7.2	49.53 ± 7.5	49.16 ± 9.1	= 0.087^*^
TGD	122.46 ± 19.1	133.11 ± 21.3	134.76 ± 23.9	= 0.129^*^
Total Cholesterol	168.74 ± 18.5	170.55 ± 25.1	168.39 ± 21.6	= 0.898^*^
ALT	52.63 ± 4.9	53.71 ± 4.7	49.56 ± 2.4	= 0.468^*^
AST	129.35 ± 15.2	140.60 ± 15.5	128.10 ± 13.5	= 0.803^*^
S. Creatinine	0.85 ± 0.03	0.86 ± 0.03	0.87 ± 0.04	= 0.901^*^
CK-MB	213.09 ± 17.9	247.23 ± 19.5	270.73 ± 23.1	**=** 0.031^*^

* *ANOVA test was used to compare the mean difference between groups*.

** *Chi-square test was used to compare proportions between groups*.

*FBG, fasting blood glucose; LDL, low density lipoprotein; HDL, high density lipoprotein; TGD, triglycerides; ALT, alanine transaminase; AST, aspartate transaminase, Creatinine serum creatinine; CK-MB, Creatine kinase. Bold values mean statistically significant*.

### Angiographic Data Correlates of Homeostasis Model Assessment—Estimated Insulin Resistance

**Figure 2:**
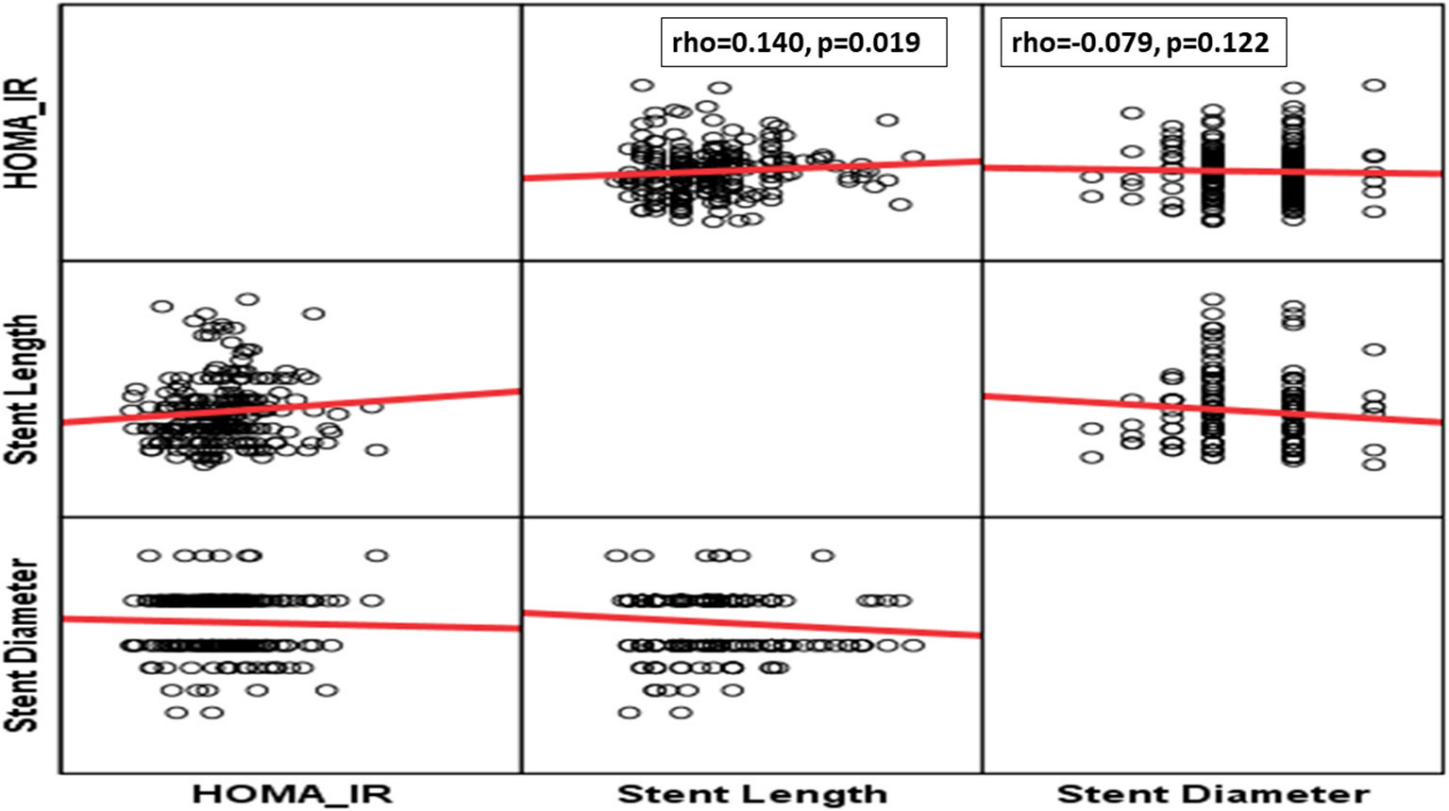
Correlation between estimated insulin resistance (HOMA-IR) and stent parameters.

Although, patients with anterior wall infarction (AWI) had insignificantly higher HOMA-IR levels, those with higher HOMA-IR experienced significantly low-grade baseline TIMI as higher tertile was founded on grade 0 baseline TIMI (Table 2). Also, patients who need longer stents experienced significantly higher levels of HOMA-IR levels (Table 2 and Figure 2). However, no differences could be detected regarding stent diameter, presence of multivessel disease, and use of DES (Table 2 and Figure 2).

**Table 2:** Angiographic data correlates of HOMA_IR.

**Parameter**	**Low** ** (*n* = 80)**	**Intermediate** ** (*n* = 80)**	**High** ** (*n* = 80)**	***P*-value**
AWI	44 (55%)	40 (50%)	52 (65%)	= 0.150^*^
Baseline TIMI				**=** 0.031^**^
•0	66 (82.5%)	67 (83.8%)	70 (87.4%)	
•1	3 (3.8%)	5 (6.3%)	8 (10%)	
•2	9 (11.3%)	3 (3.8%)	1 (1.3%)	
•3	2 (2.5%)	5 (6.3%)	1 (1.3%)	
Multi-vessel Dis.	27 (33.8%)	31 (38.8%)	28 (35%)	= 0.790^*^
Use of Stent	75 (93.8%)	74 (92.5%)	72 (90%)	= 0.670^*^
Type of Stent				= 0.858^*^
•BMS	58 (77.3%)	58 (78.4%)	54 (76.1%)	
•DES	17 (22.7%)	16 (21.6%)	17 (23.9%)	
Stent Length	26.75 ± 8.1	29.55 ± 9.3	29.93 ± 9.4	**=** 0.045^***^
Stent Diameter	3.28 ± 0.3	3.21 ± 0.3	3.29 ± 0.3	= 0.393^***^

* *Chi-square test was used to compare proportions between groups*.

** *Monte-Carlo Exact test was used to compare proportions between groups*.

*** *ANOVA test was used to compare the mean difference between groups*.

*AWI, anterior wall infarction; TIMI, thrombolysis in myocardial infarction score; BMS, bare metal stent; DES, drug eluted stent Dis disease*.

*Bold values mean statistically significant*.

### Perfusion Parameters Data Correlates of Homeostasis Model Assessment—Estimated Insulin Resistance

**Figure 3:**
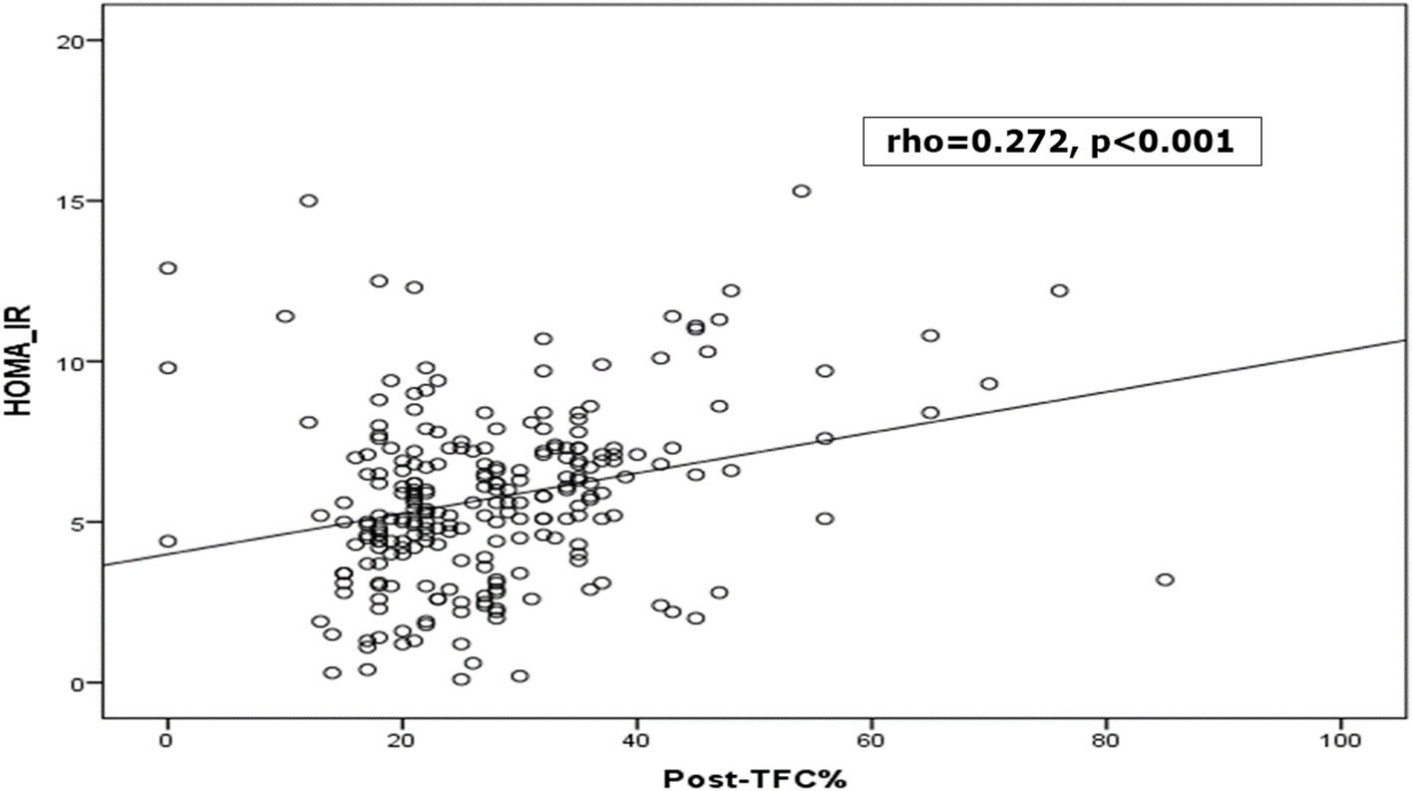
Correlation between HOMA-IR and thrombolysis in myocardial infarction (TIMI) frame count (TFC) %.

It was found that patients with MBG 0, 1, 2 had higher HOMA-IR level than those with grade 3, and those who had unchanged ST segment in their ECG after reperfusion had higher HOMA-IR level. Furthermore, postprocedural TIMI frame count (TFC) was longer in those with higher HOMA-IR level. Patients with low, intermediate HOMA-IR levels showed higher postprocedural TIMI flow grade than Grade 3 (Table 3 and Figure 3).

**Table 3:** Perfusion parameters data correlates of HOMA_IR.

**Parameter**	**Low** ** (*n* = 80)**	**Intermediate** ** (*n* = 80)**	**High** ** (*n* = 80)**	*P*-value^*^
MBG				**<** 0.001^**^
•0	4 (4.9%)	2 (2.5%)	22 (27.4%)	
•1	29 (36.3%)	37 (46.3%)	48 (60%)	
•2	45 (56.3%)	30 (37.5%)	20 (25%)	
•3	2 (2.5%)	1 (1.3%)	0 (0%)	
Post-TIMI				**=** 0.010^**^
•0	1 (1.3%)	0 (0%)	2 (2.5%)	
•1	1 (1.3%)	1 (1.3%)	3 (3.8%)	
•2	5 (6.3%)	2 (2.5%)	14 (17.5%)	
•3	73 (91.3%)	61 (67.2%)	77 (96.3%)	
S-T Resolution				**=** 0.035^*^
•Improved	63 (78.7%)	50 (62.5%)	46 (57.5%)	
•Normalized	5 (6.3%)	6 (7.5%)	6 (7.5%)	
•Unchanged	12 (15%)	24 (30%)	28 (35%)	
Post- TFC%	24.48 ± 6.4	26.64 ± 7.1	31.79 ± 8.4	**<** 0.001^***^

* *Chi-square test was used to compare proportions between groups*.

** *Monte-Carlo Exact test used to compare between groups*.

*** *ANOVA test was used to compare the mean difference between groups*.

*MBG, myocardial blush grade; post-TIMI, post interventional TIMI; S-T ST, segment; Post-TFC, post interventional TIMI frame count*.

*Bold values mean statistically significant*.

### Homeostasis Model Assessment—Estimated Insulin Resistance in Relation With Disease Outcome

A higher level of HOMA-IR was detected in those with post MI pulmonary edema; however, prolonged hospital stays, arrhythmias, LVF, cardiogenic shock, re-infarction, and early stent thrombosis complicated higher HOMA-IR level but with insignificant difference (Table 4).

**Table 4:** HOMA_IR in relation with short and long disease outcomes.

**Parameter**	**Low (*n*=80)**	**Intermediate (*n* = 80)**	**High (*n* = 80)**	***P*-value**
Hospital Stay/day	2.59 ± 0.2	2.33 ± 0.1	2.64 ± 0.1	= 0.200^*^
Arrhythmia	12 (15%)	17 (21.3%)	10 (12.5%)	= 0.303^**^
LVF	9 (11.3%)	6 (7.5%)	14 (17.5%)	= 0.146^**^
Pulmonary Edema	0 (0%)	0 (0%)	3 (3.8%)	**=** 0.013^***^
Cardiogenic Shock	3 (3.8%)	0 (0%)	1 (1.3%)	= 0.094^***^
Re-Infarction	0 (0%)	2 (2.5%)	2 (2.5%)	= 0.208^***^
Stent Thrombosis	1 (1.3%)	3 (3.8%)	2 (2.5%)	= 0.623^***^
Post pPCI EF%	53.12 ± 8.6	50.91 ± 8.7	49.33 ± 9.6	**=** 0.031^*^

* *ANOVA test was used to compare the mean difference between groups*.

** *Chi-square test was used to compare proportions between groups*.

*** *Monte-Carlo Exact test to compare proportions between groups*.

*LVF, left ventricular failure; EF, ejection fraction; pPCI, primary percutaneous coronary intervention*.

*Bold values mean statistically significant*.

### Predictors of Myocardial Blush (No/Minimal MB) Among Patients

For final multivariate regression analysis model and after adjusting for age and sex, there were 10 predictors of no/minimal myocardial blush in the studied cohort (previous CAD, hypertension, LVF, use of GPIIb/IIIa inhibitor, stent length, postprocedural TFC %, post MI LVEF %, and CKMB level). Importantly, HOMA-IR level is considered an independent risk for decreasing myocardial blush, i.e., with one unit increase in HOMA-IR level, there was decreasing myocardial blush by 20% in univariate analysis (OR:1.2, 95% CI: 1.08–1.34, *p* = 0.001) and by 10% in the multivariate analysis (OR:1.6, 95% CI: 1.01–2.04, *p* = 0.011) (Table 5).

**Table 5:** Predictors of myocardial blush (No/Minimal MB) among patients: logistic regression model.

**Variable**	**Univariate**		**Multivariate**	
	**OR (95% CI)**	***P*-value**	**HR (95% CI)**	***P*-value**
Age/years	**1.029 (1.005–1.053)**	**=** **0.017**	1.033 (0.976–1.093)	= 0.261
Sex (Male)	**1.253 (1.036–3.683)**	**=** **0.039**	1.164 (0.477–3.812)	= 0.153
Smoker	**1.309 (1.099–4.024)**	**=** **0.020**		
BMI (Obese)	0.894 (0.629–1.270)	= 0.531		
Pain-Balloon Time/h	**1.086 (1.004–1.174)**	**=** **0.040**		
Previous CAD	**3.119 (1.224–7.946)**	**=** **0.017**	**2.813 (1.195–5.001)**	**=** **0.031**
Hypertensive	**1.941 (1.013–3.718)**	**=** **0.046**	**3.274 (1.043–10.274)**	**=** **0.042**
AWI	1.114 (0.663–1.875)	= 0.685		
Killip > 1	**11.789 (2.738–50.766)**	**=** **0.001**		
ALT	**1.012 (1.001–1.024)**	**=** **0.038**		
AST	**1.004 (1.001–1.006)**	**=** **0.011**		
S. Creatinine	**2.582 (1.104–7.102)**	**=** **0.016**		
Hospital Stay/days	1.197 (0.903–1.585)	= 0.211		
LVF	**11.270 (2.613–48.606)**	**=** **0.001**	**5.157 (1.957–51.134)**	**=** **0.024**
GP IIb/IIIa Inhibitor	**5.644 (3.041–10.475)**	**<** **0.001**	**4.905 (1.649–14.586)**	**=** **0.004**
Multi-vessel Affection	**2.199 (1.253–3.860)**	**=** **0.006**	**2.602 (1.001–6.770)**	**=** **0.049**
Baseline TIMI	**0.666 (0.461–0.964)**	**=** **0.031**		
Use of Stent	0.360 (0.116–1.121)	= 0.078		
Stent Length	**1.049 (1.015–1.085)**	**=** **0.005**	**1.066 (1.009–1.127)**	**=** **0.023**
Post PCI TFC %	**1.229 (1.162–1.300)**	**<** **0.001**	**1.328 (1.216–1.451)**	**<** **0.001**
Post PCI LVEF%	**0.931 (0.899–0.963)**	**<** **0.001**	**0.946 (0.840–0.979)**	**=** **0.002**
Unchanged ST Resolution	**2.143 (1.517–3.028)**	**<** **0.001**		
CK-MB	**1.003 (1.001–1.005)**	**=** **0.001**	**1.009 (1.002–1.084)**	**=** **0.034**
One-month LVEF%	**0.885 (0.851–0.920)**	**<** **0.001**		
HOMA_IR	**1.202 (1.079–1.339)**	**=** **0.001**	**1.156 (1.009–2.039)**	**=** **0.011**

*zOR = odds ratio; CI, confidence interval*.

*BMI, body mass index; CAD, coronary artery disease; AWI, anterior wall infarction; ALT, alanine transaminase; AST, aspartate transaminase; LVF, left ventricular failure; TIMI, the thrombolysis in myocardial infarction; TFC TIMI, frame count; LVEF, left ventricle ejection fraction; ST, st segment; CK-MB, creatine kinase MB; HOMA-IR, homeostatic model assessment of insulin resistance*.

*Bold values mean statistically significant*.

## Discussion

The present study analyzed IR in the acute phase of STEMI in non-diabetic patients treated by pPCI. First, it confirmed that acute IR in the early post pPCI period is common even in non-diabetic patients; second, IR, as estimated by HOMA-IR, affects myocardial perfusion especially MBG; and third, IR was related to the in-hospital MACE.

IR in the early phase of STEMI is considered part of the acute glycol-metabolic response to stress (21) Generally, in critically ill patients, acute IR is related to more severe acute illness and leads to poor clinical outcome (22, 23). The mean HOMA-IR level in this study was beyond the normal range as defined by a substantial number of epidemiological studies (24, 25). The majority of patients were obese and have significantly earlier post PCI hyperinsulinemia and, hence, higher HOMA-IR tertile. The same increment in insulin in the early post PCI period in non-diabetic patients was previously reported (26); Nishio et al. did serial HOMA index measurements among patients who underwent pPCI and identified those with transient IR, in whom HOMA index correlated with stress hormones (catecholamine and cortisol) and patients with persistent IR, in whom HOMA index during follow-up correlated with leptin and contributed to stent restenosis (27).

More cardiac necrosis was reported in those with higher IR. The relationship between acute IR in non-diabetic STEMI patients and myocardial damage in terms of peak enzymes was previously reported (28, 29). In this study, both HOMA indices were correlated well with the peak CKMB in the presence or absence of other confounders and insignificantly higher insulin resistance among those with anterior wall STEMI.

Acute hyperglycemia and IR *per se* predicted impaired epicardial flow before pPCI (30). This matched with our study findings, which speculated that IR was significantly higher in those with grade 0 TIMI either at baseline or post-interventional, and this may explain why those with higher HOMA index needed significantly longer stents. Also, there was significantly higher IR in those with more postprocedural TFC%. It may be related to the increased oxidative stress in STEMI, increased vascular cell apoptosis, and hence induced acute endothelial dysfunction.

The relationship between ST-R and acute IR was not conclusive. Acute hyperglycemia was related to limited ST-R in mixed diabetic and non-diabetic STEMI population after thrombolytic therapy (31) and after pPCI (32) and was identified as a predictor of ST resolution after pPCI. In the current study, in patients without diabetes, incomplete ST resolution was significantly more frequent among those with acute IR. Although the inverse relationship between HOMA IR level and myocardial perfusion was described in different states; to the best of our knowledge the current study is the first to link acute IR assessed by the HOMA index and myocardial blush in non-diabetic STEMI patients to assess microvascular perfusion.

Moreover, it was found that acute IR significantly affect myocardial blush; higher HOMA-IR level was associated with lower MBG and vice versa. Higher levels of HOMA-IR either alone or after adjusting for all other confounders (hypertension, previous CAD, LVF, multivessel CAD, reduced LVEF, and increased CPK_MB level) were considered important predictors of markedly reduced myocardial blush. This may be explained by IR in the setting of STIMI being associated with poor myocardial reperfusion, impaired coronary microcirculation (33) and collateralization (34) and reduced collagen deposition in the scar (35). These factors potentially lead to greater infarct size, post-infarction LV dilation, and finally a higher incidence of heart failure (36). Nevertheless, the implicated mechanisms await precise characterization in future studies. It was speculated in this research that acute IR was associated with poorer outcome, as higher HOMA-IR tertile was significantly observed in those with pulmonary edema and reduced LVEF. On the other hand, IR was insignificantly higher in those with LVF, prolonged hospital stays, arrhythmia, stent thrombosis, re-infarction, and cardiogenic shock.

## Conclusion

The current study revealed that acute IR was prevalent in non-diabetic patients with STEMI and was identified as an independent predictor of post infarction myocardial and microvascular injury. Likewise, it was found to be associated with poor in-hospital outcome.

## Data Availability Statement

The raw data supporting the conclusions of this article will be made available by the authors, without undue reservation.

## Ethics Statement

The studies involving human participants were reviewed and approved by prof/Mahmoud abdel-Aleem- Medical ethics committee IRB no.17300510. The patients/participants provided their written informed consent to participate in this study. Written informed consent was obtained from the individual(s) for the publication of any potentially identifiable images or data included in this article.

## Author Contributions

SK and MA conceptualized and designed the study, conducted a literature search, conducted the clinical studies, prepared the experimental study manuscript, and edited and reviewed the article. AH was in charge of the definition of intellectual content, literature search and manuscript review, clinical studies, experimental studies, and data acquisition. GS conducted the clinical studies, experimental studies, and acquired the data. All authors contributed to the article and approved the submitted version.

## Conflict of Interest

The authors declare that the research was conducted in the absence of any commercial or financial relationships that could be construed as a potential conflict of interest.

## Abbreviations

CMF: coronary microcirculatory function
STEMI: ST-segment elevation myocardial infarction
pPCI: primary percutaneous coronary intervention
STR: ST segment resolution
HOMA-IR: homeostatic modeling assessment—insulin resistance
AMI: acute myocardial infarction
MBG: myocardial blush grade
MACE: major adverse cardiovascular events.
